# Tough Art and Microbial Drama

**DOI:** 10.3201/eid1801.AC1801

**Published:** 2012-01

**Authors:** Polyxeni Potter

**Affiliations:** Centers for Disease Control and Prevention, Atlanta, Georgia, USA

**Keywords:** art science connection, emerging infectious diseases, art and medicine, tough art and microbial drama, Dioskurides of Samos, Musici Ambulanti, Hellenistic drama, mosaic, dengue, brucellosis, about the cover

**Figure:**
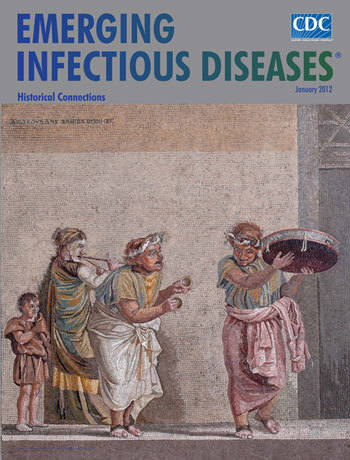
**Dioskurides of Samos, *Musici Ambulanti*, Mosaic (c. 2200 years ago) (41 cm × 43 cm).** Museo Archeologico Nazionale, Piazza Museo Nazionale, Napoli, Italy

Glass, ceramic, marble or limestone, shells, pebbles, enamel, ivory, mother-of-pearl, gold, painted and semiprecious stones―in the most refined form especially cut into hard cubes known as *tesserae―*were set at different angles and depths and arranged into tactile patterns depicting mythologic subjects, everyday or theatrical scenes, and many other themes to adorn architectural structures. Even though they were sometimes found outside buildings, mosaics were primarily used on interior surfaces. As the practice became more common, *tesserae* of colored glass were expressly produced to provide intense blue, red, and green hues, “Like very rubies from gold patinas gleaming,” as Dante Alighieri would write in the Divine Comedy when he viewed the ancient mosaics in Ravenna, Italy.

Surface decoration made of small particles set in a foundation to form a unified whole likely began as assembled colored stones as far back as the third millennium BCE. Organizing these stones into patterns, or mosaic making, became an art form and flourished in antiquity, and because of its structural strength and durability, survived on the floors and walls of ancient palaces, cathedrals, and affluent homes inspiring new technical and artistic greatness throughout history and continuing to thrive as monumental decoration in the 19th and 20th centuries.

As art form, the mosaic offers pure color expression through the setting (*opus*) of individual fragments into clay, plaster, or mortar. The laborious process relies on materials that, unlike brushstrokes, are inherently inflexible. Because the final image is meant to be viewed from a distance, color gradation, an optical illusion, is achieved in the eyes of the viewer. The *tesserae* vary in size and shape―small stones for faces and hands, larger ones for backgrounds―and allow for precision in laying the mosaics and for special visual effects. They are tilted and spaced irregularly to create glitter and refract natural light. They capture, reflect, absorb, and splinter the light which, multiplied as with prisms, breaks into an infinite number of chromatic units. Or, as an unknown poet put it also in reference to the Ravenna mosaics, “Either light was born or imprisoned here. It reigns supreme.”

*Musici Ambulanti*, on this month’s cover, found in the ruins of a structure known as the Villa of Cicero near the city of Pompeii, is one of the finest examples of ancient mosaic making. Signed by Dioskurides of Samos, known to us only from mosaics bearing his name, this work has been linked to images found more than 3 centuries later in the House of Menander in Mytilene. These mosaics contain scenes from the plays of Menander, most famous of the writers of Attic New Comedy in the second half of the 4th century BCE. He was known for portraying ordinary people and their lives. The Dioskurides panel roughly resembles a mosaic scene in Mytilene that likely originated from an illustrated manuscript or a painting of Menander’s play *Theophoroumene* (The Girl Possessed).

Only fragments of the play remain, so the story line is sketchy. The heroine was possessed by Kybele, mother of the gods and mistress of nature, whose cult was associated with ecstatic states and dances performed to drums or cymbals. Illustrations of the play have become a visual record of Greek drama as well as of painting and music in the Hellenistic period.

In the Dioskurides panel, three actors wearing masks are performing as musicians. The figures are individualized through their actions and postures. A man on the right plays the tympanon. Another man with cymbals, in the center, is turned toward the tympanon player. And a woman with a *hetaera* (courtesan) mask, playing the aulos (a reed instrument) is accompanied by an assistant, an unmasked boy on the left. The actors’ bodies cast shadows on the wall. The scene brims with hilarity and easy, flowing movement.

The image is architectural, with columns supporting a coffered roof. Pillars on the side extend to the top. Central figure representations are contained in marble frames. The background is simple, mainly neutral bands against which the figures stand out and a street with a house door behind them. The mosaic is made with minute glass *tesserae* (none larger than 0.25 cm, in many parts of the figures smaller than 1 mm square) in *opus vermiculatum* (worm-like), a technique in which one or more rows of *tesserae* curve around the figures, emphasizing them and foreground elements in a halo effect. As in many of the best mosaics of the period, the mortar is tinted to match the *tesserae*. In this case, the mortar was painted over with a very fine brush after the *tesserae* were set. The colors in the tunics of the men and the aulos player vary in tone to produce shimmering light.

The term “mosaic,” derived from the Greek *Mouseios* (of the muses), has come to mean anything made in mosaic style and can apply to literary and other compositions outside art. So filled with possibilities for metaphorical interpretation, the term has been widely used, most famously in regards to multiculturalism. An ancient form of art become a common modern metaphor, the mosaic has shown remarkable resilience quite apart from its concrete solidity of structure.

“Painting in stone,” as mosaic making has sometimes been called, is painstaking and technically demanding, particularly in its inspired form, which combines seamlessly and in complete balance color, light, and rhythm. Yet it has persisted through the ages, its tiny ingredients refined and enriched but otherwise essentially the same. The lyrical scene *Musici Ambulanti* with its ancient humor and contemporary perspective opens up the topic for metaphorical discussion. Here is a medium that owes its perseverance to its inflexibility.

In this issue, along with current reports on influenza, prion disease, and multidrug-resistant tuberculosis, we offer some historical reports, reminders that tough art is not all that survives the ravages of time, persisting through the ages. Pathogenic agents do too. This is why we still speculate on the plague of Thebes and the possibility, among many others examined, that brucellosis was the culprit. Brucellosis, a highly transmissible zoonosis, remains endemic to the Mediterranean basin. This is too why we read with interest about a dengue fever epidemic in Athens viewed through a daily newspaper in 1927–1931. Dengue fever, also an ancient scourge of global proportions, still causes outbreaks in tropical areas around the globe and sometimes even in Florida.

Unlike mosaics, which owe their resilience to the inflexibility of their medium, microbes often owe their persistence to extraordinary flexibility brought about by genetic plasticity and a short life cycle. When their genes divide, the offspring are unlike their parents in ways that may make them survive or cause disease more effectively. They adapt quickly and remain with us throughout the ages. Like the acting musicians in Dioskurides’ mosaic, they play and they dance to their own rhythm, compelling, often masked, and impervious to the march of time.
